# Edge detection in microscopy images using curvelets

**DOI:** 10.1186/1471-2105-10-75

**Published:** 2009-03-03

**Authors:** Tobias Gebäck, Petros Koumoutsakos

**Affiliations:** 1 Chair of Computational Science, ETH Zürich, Universitätstrasse 6, CAB H69.2, ETH Zürich, CH-8092 Zürich, Switzerland

## Abstract

**Background:**

Despite significant progress in imaging technologies, the efficient detection of edges and elongated features in images of intracellular and multicellular structures acquired using light or electron microscopy is a challenging and time consuming task in many laboratories.

**Results:**

We present a novel method, based on the discrete curvelet transform, to extract a directional field from the image that indicates the location and direction of the edges. This directional field is then processed using the non-maximal suppression and thresholding steps of the Canny algorithm to trace along the edges and mark them. Optionally, the edges may then be extended along the directions given by the curvelets to provide a more connected edge map. We compare our scheme to the Canny edge detector and an edge detector based on Gabor filters, and show that our scheme performs better in detecting larger, elongated structures possibly composed of several step or ridge edges.

**Conclusion:**

The proposed curvelet based edge detection is a novel and competitive approach for imaging problems. We expect that the methodology and the accompanying software will facilitate and improve edge detection in images available using light or electron microscopy.

## Background

The analysis of microscopy images is a time consuming task in many microbiological and biomedical laboratories. There is an ever-increasing need for analyzing large numbers of images acquired with microscopes in connection with different assays, where one wishes to measure the number of cells, the size of certain objects, the area occupied by cells, etc [1]. In recent years a number of software tools, such as ImageJ (National Institutes of Health), CellProfiler [2], NeuroLucida (MicroBrightField, Inc.), etc., have been developed to facilitate some of these tasks. Nevertheless, many of the tasks are still performed manually, and there is a great need for accurate and reliable software that can automate the image analysis and thus increase the throughput in these assays.

The problem of edge detection has a long history in computer vision (see e.g. [3] and references therein). The simplest edge detection schemes compute the approximate gradient of the intensity map of the image by applying a filter, such as the Sobel, Prewitt or Roberts filter, and then use a thresholding to extract the edges identified as areas with large gradient. Other methods use the second derivatives of images and search for zero-crossings instead of maxima. More sophisticated edge detectors such as the one developed by [4] use the intensity gradient, after it has been appropriately smoothed, search for local maxima only in the gradient direction, and apply additionally a 'hysteresis thresholding' to maximize the edge connectivity. These methods however encounter problems with images available from electron or light microscopy, as they are rather sensitive to noise and when smoothing is applied to reduce the noise, the edges also get smoothed to the extent that they cannot be detected.

A number of edge detection methods employ 2D Gabor filters. These filters are characterized by frequency, width and direction and have been mainly applied to object recognition problems. [5] introduced an edge detection scheme using Gabor filters, and derived optimal design parameters for detection of step edges. Gabor filters have also been applied by [6] to detect grain boundaries in electron microscopy images of metals and alloys. In many ways, 2D Gabor filters are similar to curvelets, and we will discuss them in more detail below.

Other powerful edge detection methods are also available, such as 'snakes' or 'active contours', which also use gradient information from the image to evolve a connected contour that minimizes its 'energy' in the landscape defined by the image [7,8]. These methods successfully detect boundaries of objects with an intensity (or pattern) difference compared to the background, but in microscopy images it is often the case that the interior and the exterior of the object of interest show no difference in intensity.

The curvelet transform was developed by [9] in order to provide a multiscale representation which improves on the wavelet transforms in representing edges in an image. In fact, it has been shown that curvelets provide a near-optimal representation of *C*^2^-edges, considering the number of curvelet coefficients needed to represent the edge to a given accuracy [9]. This property makes curvelets useful for denoising of images with edges [10,11], but also, as we shall see, makes them useful for edge detection and extraction. With the development of the fast discrete curvelet transform [10], and the freely available implementation CurveLab , the curvelet transform is gaining recognition as a potent image analysis method with applications ranging from medical imaging [12] to fluid mechanics [13]. A thorough comparison of the curvelet transform with wavelets and ridglets has demonstrated the capabilities of this method in texture classification of images obtained by Computed Tomography (CT) scans [12].

The present edge detection scheme uses the discrete curvelet transform to extract information about directionality and magnitude of features in the image at selected levels of detail. The edges are then extracted using the 'non-maximal suppression' and 'hysteresis thresholding' steps of the Canny algorithm [4]. The directional information from the curvelets is then further used to connect edge segments that were erroneously separated. The scheme is successful in detecting elongated structures in the images, such as for example membranes. Unlike for example the Canny algorithm, the edges detected by our scheme are not detected on the single pixel level, but their width is determined by the choice of curvelet detail levels specified in the analysis. The edges we detect are not necessarily step edges between regions of high and low intensity. The curvelet transform enables a multilevel decomposition of the image so that the magnitudes of the curvelet coefficients signify intensity variations on their level of detail. Hence, edges are detected when intensity variations are large on a selected scale. This is useful in detecting for example membranes in microscopy images, since they show up as several parallel edges.

The paper is organised as follows: in the next section, we give a brief introduction to the curvelet transform, as well as a description of the Canny edge detector and the Gabor filter edge detector of [5]. In the Method section, we present our edge detection scheme in detail and in the Results section we apply our scheme to two microscopy images, comparing the results to the results achieved using the Canny and Gabor filter edge detection schemes. The last section summarizes and discusses our results.

### The discrete curvelet transform

The discrete curvelet transform was introduced by [10] in two forms, the wrapping version and the unequally spaced FFT (USFFT) version. Since the wrapping version is faster and invertible up to numerical precision, while the USFFT version is only approximately invertible, we use only the wrapping version throughout this paper. We note however that the introduction below applies in most parts to both versions.

We introduce the discrete curvelet transform applied to an image with intensity values given by the function *f*(*x*_1_, *x*_2_), *x*_1 _= 0, 1,..., *N*_1 _- 1, *x*_2 _= 0, 1,..., *N*_2 _- 1, whose discrete Fourier transform (DFT) is

$$\hat{f}(n_{1},n_{2})=\sum_{x_{2}=0}^{N_{2}-1}\sum_{x_{1}=0}^{N_{1}-1}f(x_{1},x_{2})e^{-2\pi i(n_{1}x_{1}/N_{1}+n_{2}x_{2}/N_{2})}.$$

The discrete curvelet transform is now a decomposition of the image *f *into the curvelet coefficients *c*_*jlk*_, such that

$$f(x_{1},x_{2})=\sum_{j=1}^{J}\sum_{l=0}^{L_{j}-1}\sum_{k_{1}=0}^{K_{jl,1}-1}\sum_{k_{2}=0}^{K_{jl,2}-1}c_{jlk}\varphi_{jlk}(x_{1},x_{2}),$$

where *k *= (*k*_1_, *k*_2_) and *φ*_*jlk *_is the curvelet on level *j *with direction *l *and spatial shift *k*. Additionally, the curvelet transform preserves *l*^2^-norms, i.e.

$$\sum_{j,l,k}|c_{jlk}{|}^{2}=\sum_{x_{1},x_{2}}+|f(x_{1},x_{2}){|}^{2}.$$

The discrete curvelet transform thus provides a decomposition of the image *f *into *J *detail levels, with *L*_*j *_directions on each level, and *K*_*jl*,1 _× *K*_*jl*,2 _spatial shifts for each of these directions. The curvelet *φ*_*jlk *_is defined through its discrete Fourier transform as

$${\hat{\varphi }}_{j0k}(n_{1},n_{2})=U_{j}(n_{1},n_{2})e^{-2\pi i(k_{1}n_{1}/K_{j0,1}+k_{2}n_{2}/K_{j0,2})}$$

and

$${\hat{\varphi }}_{jlk}=S_{\theta_{l}}^{T}{\hat{\varphi }}_{j0k}.$$

Here, $S_{\theta_{l}}$ is a shearing matrix, which shears the grid on which the curvelet is evaluated by an angle *θ*_*l*_. The slopes defined by the angles *θ*_*l *_are equispaced. *U*_*j *_is a frequency window function with compact support, which is approximately 1 inside a wedge around the *n*_1_-axis with 2^*j *^≤ *n*_1 _< 2^*j*+1^, and decreases quickly to zero outside this area. The *U*_*j *_are defined so that

$$\sum_{j}\sum_{l}|[S_{\theta_{l}}U_{j}](n_{1},n_{2}){|}^{2}=1.$$

Thus, the discrete curvelet transform provides a decomposition of the frequency space into dyadic rectangular coronae, each of which is divided into wedges, the number of which doubles with every second level.

**Figure 1:**
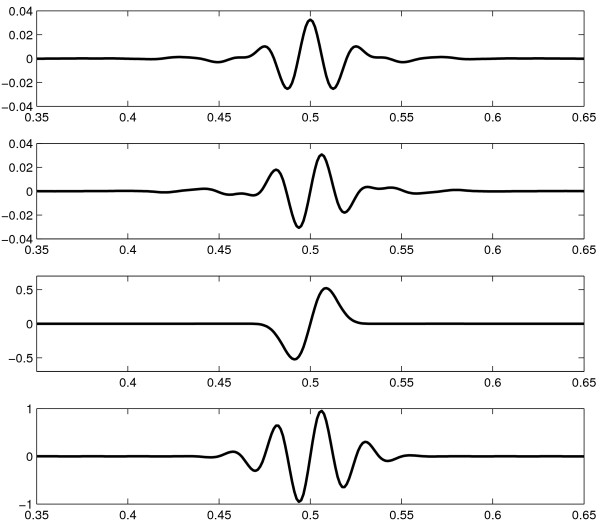
**Curvelet and Gabor filter profiles**. Profiles along the oscillatory direction of curvelets and Gabor filters. From top to bottom: real part of a curvelet; imaginary part of a curvelet; an odd Gabor filter with *σ*·*ω *= 1; an odd Gabor filter with *σ*·*ω *= 4. The imaginary part of a curvelet is similar to an odd Gabor filter with *σ*·*ω *= 4, but has a different decay.

In figure 1, the top two graphs show respectively the profiles along the oscillating directions of the real and imaginary parts, of a curvelet *φ*_*jlk*_. The imaginary part is odd with respect to its center, while the real part is even.

The curvelet coefficients *c*_*jlk *_are computed as

$$c_{jlk}=\langle \hat{f},{\hat{\varphi }}_{jlk}\rangle .$$

This scalar product is computed as a sum over a parallelogram containing the support of *φ*_*jlk*_, which in the wrapping version is wrapped by periodicity onto a rectangular area near the origin, namely 0 ≤ *n*_1 _<*K*_*jl*,1_, 0 ≤ *n*_2 _<*K*_*jl*,2_. The size of this rectangle also determines the ranges for the spatial shifts *k*_1 _and *k*_2_, and therefore the resolution at curvelet level *j*.

The curvelet transform can be seen as an efficient computation of convolutions with filters with profiles as in figure 1, for a range of different scales and directions, evaluated at discrete points on grids adapted to the size of the filter. Because of the scaling of the frequency window *U*_*j*_, the curvelet profile has the same shape at all scales.

On the coarsest level, *j *= 1, the curvelets are non-directional (i.e. *L*_1 _= 1) and are similar to the Meyer wavelet scaling function (see e.g. [14]), and on the finest level, *j *= *J*, a choice is given in the implementation in CurveLab to use curvelets (with a directional decomposition) or wavelets (with no directional information). If curvelets are used on the finest level, they may be included in the edge detection procedure below like any other curvelet level. Throughout this paper, however, we have used wavelets on the finest level, because of the shorter execution time and smaller memory requirements, but also because for our examples we were not interested in directional information on the smallest scale.

### The Canny edge detector

[4] introduced an edge detection algorithm based on the idea of applying a filter to the image that is optimal in the identification of step edges, and which is defined so that the output of the filter operation will have a maximum at the location of the edge. The problem of edge detection is then reduced to finding ridges of local maxima in the filtered image. In practice, such as in the implementation of the Canny edge detector in MATLAB (The MathWorks), and as suggested by Canny, the optimal filter is approximated by the derivative of a Gaussian of variable variance. Edges of different width may then be detected by manually choosing different variances.

Since the convolution with the gradient of a function is equal to the gradient of the convolution, the filtering can be efficiently performed by first convolving with a Gaussian to smooth the image and then computing the gradient. The extraction of ridges of maxima is performed by looking for local maxima in the gradient direction. Additionally, the edge pixels are thresholded using two thresholds in order to reduce 'streaking', that is the subdivision of edges into short segments, while simultaneously reducing the probability to extract isolated edge points.

An implementation of the Canny edge detector thus amounts to the following steps (see also [[3], chapter 10]):

Algorithm 1 (The Canny edge detector)

*1. Smooth the image by convolving with a Gaussian of variance σ^2^*.

*2. Compute the gradient of the smoothed image, and compute its magnitude and direction*.

*3. Non-maximal suppression: Select the pixels where the gradient magnitude has a local maximum in the direction of the gradient*.

*4. Using two specified thresholds, T*_1_* and T*_2_, *with T*_1 _<*T*_2_, *mark selected pixels with gradient magnitude larger than T*_2_* as 'strong', and pixels with magnitude between T*_1_* and T*_2_* as 'weak'*.

*5. Select all strong pixels, and all weak pixels that are connected to strong pixels horizontally, vertically or diagonally*.

It is interesting to note that [4] emphasizes the need for multiple widths (or scales) of the filter, as well as different orientation, in order to detect all the edges. These are precisely the two features provided by curvelets.

### Gabor filters

A 2D odd Gabor filter, characterized by the angular frequency *ω*, the width *σ *and the direction angle *θ*, is given by

(1) $$s(x,y)=\exp\left(-\frac{x^{2}+y^{2}}{2\sigma^{2}}\right)\sin(\omega (x\cos\theta +y\sin\theta )).$$

The 1D profile of this filter (along the direction given by *θ*) for two different choices of *σ *and *ω *is shown in figure 1. There is also an even Gabor filter, where the sine in equation (1) is replaced by a cosine, and the two can be combined as real and imaginary parts of a complex filter. As it is seen in figure 1, the Gabor filters have a profile similar to the one of curvelets. The main difference is that the parameters *ω*, *σ *and *θ *may be chosen freely for Gabor filters, while they are fixed to discrete values for the curvelets, with the frequency and width given by the curvelet level and the choice of directions given by the partition of the frequency space. Thus, the Gabor filters are more flexible, which is both an advantage and a disadvantage. The advantage is obviously that the parameters may be chosen to optimize performance for different applications, while the disadvantage is that the generality makes it difficult to find a set of parameters that actually works, making the filters more complicated to use.

Another significant difference is that the even Gabor filter does not have zero integral, and it is thus sensitive to absolute intensity values, not only variations in intensity. This makes it less suitable for edge detection applications. For curvelets, however, both the real and imaginary parts can be used to detect different types of edges, which we make use of in our scheme.

[5] argue that the choice of *σ *and *ω *in equation (1) so that *σ*·*ω *= 1 is optimal for detection of step edges by convolution with odd Gabor filters. With this choice, the Gabor filter is very similar to the derivative of a Gaussian used in the Canny algorithm, and it is therefore not surprising that this choice gives good results for step edges. To select the angle *θ*, they suggested estimating the edge direction at each point of an image from the gradient of the smoothed image and then evaluate the convolution with a Gabor filter using the gradient direction for *θ*. The edges were then marked as pixels of local maxima in filter response. This method is used in the examples in the Results section.

[6] used a combination of even and odd Gabor filters of different scales, and with *σ*·*ω *≈ 1.25, to detect grain boundaries in electron microscopy images of metals and alloys. They were able to detect edges with a wide range of characteristics, both step and ridge edges, and of different widths, but the sizes of the filters had to be tuned manually to the images, and as mentioned above the even Gabor filter is sensitive to background intensity differences.

## Methods

In this paper we combine the curvelet transform with a Canny edge detector algorithm leading to an edge detection scheme consisting of the following steps:

Algorithm 2 (Edge detection using curvelets)

1. Apply the fast discrete curvelet transform to the image

*2. Using one or several levels of curvelet coefficients, extract the maximum magnitude and direction at each location on the finest of the chosen levels of curvelets. This gives the directional field of the edges in the image*.

*3. Apply steps 3 to 5 of the Canny edge detector (algorithm 1), with the directional field as input, to extract the edges*.

*4. Extend the extracted edges along the directional field computed in step 2, to connect neighboring edge segments*.

*5. Map the edges onto the original image and perform post-processing suitable to the application*.

We implemented the scheme in MATLAB (The MathWorks) using CurveLab  for the fast discrete curvelet transform [see Additional file 1]. The steps of the scheme will now be discussed in more detail.

### Applying the fast discrete curvelet transform

We apply the fast discrete curvelet transform, in the 'wrapping' version, with the default values for number of levels and directions. We use wavelets at the finest level since it makes the transform faster, and since the objects we are interested in live on coarser levels, so directional information at the finest level is of no importance. If edges on the finest level are of interest, however, curvelets can be used on the finest level and the edge detection scheme may be applied to this level as well.

### Extracting the directional field

As each curvelet coefficient *c*_*jlk *_is associated with a particular location (the index *k*) and a particular direction (the index *l*), it is easy to use the curvelet coefficients to extract a field describing the directions and locations of major features in the image by the following procedure.

We first select a number of curvelet levels {*j*_1_,..., *j*_*P*_}, depending on the size of the image features we are interested in, typically the width of the edges. The selected levels are usually determined by trial and error, but it is generally better to include more than one level, as edges may vary in width and leave traces on several levels. Each selected level, *j*_*i*_, is associated with a grid $G_{i}$ = {(*k*_1_, *k*_2_)|0 ≤ *k*_1 _<$K_{1}^{i}$, 0 ≤ *k*_2 _<$K_{2}^{i}$} of size $K_{1}^{i}\times K_{2}^{i}$, determined by the discrete curvelet transform. It is on the grid $G_{P}$ of size $K_{1}^{P}\times K_{2}^{P}$ on the finest selected level, *j*_*P*_, that we compute the directional field. Note that this grid contains fewer grid points than the original image has pixels, so the directional field is not computed for each pixel in the image.

Now, each curvelet coefficient $c_{j_{i}lk}$ on a coarser level *j*_*i *_is mapped onto a subset *A*_*i*, *P *_(*k*) of the grid $G_{P}$ by mapping it to the grid points it overlaps, treating the curvelets as centered on the grid points. That is,

$$A_{i,P}(k)=\{({k^{\prime }}_{1},{k^{\prime }}_{2})\in G_{P}|k_{d}-0.5\leq {k^{\prime }}_{d}/K_{d}^{P}\cdot K_{d}^{i}<k_{d}+0.5,d=1,2\}.$$

Similarly, each curvelet coefficient *c*_*jlk *_is associated with a direction determined by the index *l*. Since the number of directions varies with the curvelet level, with the number of directions doubling with every second level, the coefficients on coarser levels need to be mapped to all directions on the finest selected level that they overlap with. We therefore define the set *D*_*i*, *P*_(*l*) as

*D*_*i*, *P *_(*l*) = {*l*' ∈ {0,..., *L*_*P *_- 1}|*l *≤ *l*'/*L*_*P*_·*L*_*i *_<*l *+ 1}

if *L*_*i *_is the number of directions on level *j*_*i*_.

Now, for each direction *l *and location *k *= (*k*_1_, *k*_2_) on the finest level *j*_*P*_, we sum up the magnitudes of the curvelet coefficients as

$$\begin{array}{ccc} M_{lk}=\sum_{i=1}^{P}\sum_{l^{\prime }\in D_{i,P}(l)}\sum_{k^{\prime }\in A_{i,P}(k)}\left|c_{j_{i}l^{\prime }k^{\prime }}\right|, & l=0,...L_{P}/2-1, & k\in G_{P}. \end{array}$$

Note that we need only compute *M*_*lk *_for *l *up to *L*_*P*_/2-1, since the directions *l *and $\tilde{l}$ = *l *+ *L*_*P*_/2 are separated by 180 degrees and thus represent the same direction. Furthermore, if we apply the curvelet transform to a real-valued image, then $c_{j\tilde{l}k}$ is the complex conjugate of *c*_*jlk*_, so |$c_{j\tilde{l}k}$| = |*c*_*jlk*_|. It should be noted that by using the absolute values of the curvelet coefficients, we combine the even and odd filters shown in figure 1. An alternative would be to add only the real parts (the even filters) or the imaginary parts (the odd filters), but since the edges we wish to detect may vary in appearance along their length, combining the two filters gives more reliable results for our applications.

Having computed *M*_*lk*_, we can now compute the major direction *l*_0_(*k*) at each grid point by

(2) $$\begin{array}{cc} l_{0}(k)=\underset{l=0,...,L_{P}/2-1}{\arg\max}M_{lk}, & k\in G_{P}, \end{array}$$

and define the field Ψ(*k*) = (Ψ_1_(*k*), Ψ_2_(*k*)), *k *∈ $G_{P}$ as

$$\Psi (k)=(M_{l_{0}k}\cdot \cos\theta_{l_{0}},M_{l_{0}k}\cdot \sin\theta_{l_{0}}),$$

where $\theta_{l_{0}}$ is the angle associated with the direction *l*_0_, by the definition of the discrete curvelet transform. The angle *θ*_0 _is taken along the valleys of the curvelets, meaning that the direction of the field Ψ(*k*) will be along the edges in the image.

Alternatively, we may compute the total curvelet magnitude at each position by

(3) $$\begin{array}{cc} {\hat{M}}_{k}=\sum_{l=0}^{L_{P}/2-1}M_{lk}, & k\in G_{P}, \end{array}$$

which gives a non-directional measure of the intensity variations in the image to compare with the directional measure Ψ(*k*).

Before picking the maximal direction, we also have the possibility to perform some smoothing, for example by averaging neighboring values of *M*_*lk*_, in both indices. This often leads to better results in the next step of our scheme. In particular, averaging over neighboring directions tends to give more distinct edges in the curvelet magnitude image, since curvelets that are not perfectly aligned with the edge also account to some extent for the edge.

### Extracting the edges using non-maximal suppression

The directional field Ψ(*k*) computed as described above indicates the location (by the magnitude |Ψ(*k*)|) and the direction (by the angle $\theta_{l_{0}}$) of edges in the image. This is information similar to the one attained by computing the gradient of the image. Therefore, we may apply steps 3 to 5 of the Canny edge detector (algorithm 1) at this stage, replacing the steps of smoothing and computing the gradient in the original Canny scheme by the computation of the field Ψ(*k*) above.

The last three steps of the Canny algorithm will then trace along the ridges of high magnitude of Ψ(*k*), selecting pixels where |Ψ(*k*)| has a local maximum in the direction perpendicular to the edge, and is larger than one of the two thresholds, the weak and the strong threshold. The weak pixels that are connected to strong pixels are kept, while the other weak pixels are ignored, and the output is a binary image with 1's on the selected edges.

### Extending the edges along the directional field

Even if the thresholding in the Canny algorithm is designed to reduce 'streaking' (the subdivision of edges into short segments), the edges that are extracted from steps 1 to 3 of algorithm 2 are not always connected to the desired extent. The reason for this may be that the influence of nearby edges prevent some pixels from being local maxima, so that they are ruled out by the non-maximal suppression, or that some pixels happen to have a value smaller than the low threshold, even though they are actually part of the edge.

As a remedy, the edges may be extended as follows: starting at the end points of the already selected edge segments, we take a step in the direction given by the directional field, away from the edge segment we start at. We then continue moving along the directional field until a specified number of steps have been taken, or we end up on a different edge segment. If we end up on a different edge segment, the entire path we moved along is included as an edge. A threshold is also employed to exclude pixels with small magnitude to be included in the extension.

### Post-processing

The output from the scheme described so far is not always what one would like to present as a final output. In particular, one might want to make sure that the edges form a connected loop, remove isolated edge segments, and thin the edges. Furthermore, since Ψ(*k*) is not defined on the same grid as the original image, the selected edges will need to be interpolated onto the original image grid. This post-processing has to be adapted to the particular application.

## Results

### An electron microscopy image

**Figure 2:**
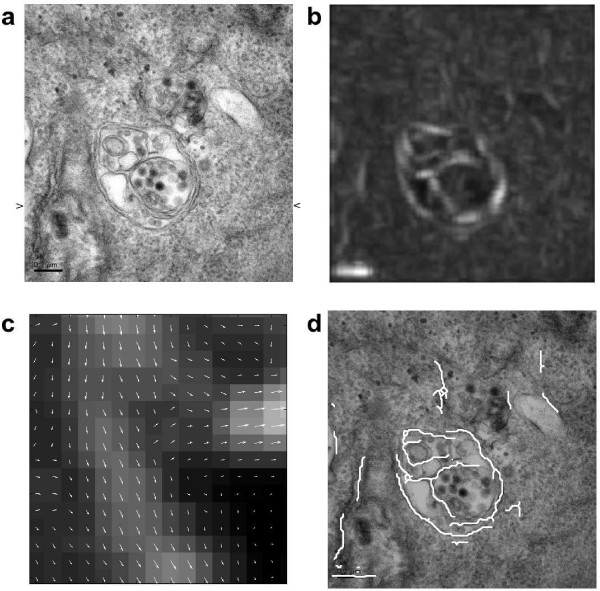
**Edge extraction on a microscopy image of a vesicle**. An electron microscopy image of a vesicle (a), to which the edge extraction scheme is applied. The magnitude of the directional field extracted from the curvelet coefficients is shown in (b), with an extract showing the direction of the field in a small part of the image shown in (c). The last image (d) shows the final result of the edge extraction overlaid on the original image, after the edges have been extracted using the non-maximal suppression, and extended along the directional field to connect the different edge segments. Image courtesy of Prof. Urs Greber, University of Zürich.

**Figure 3:**
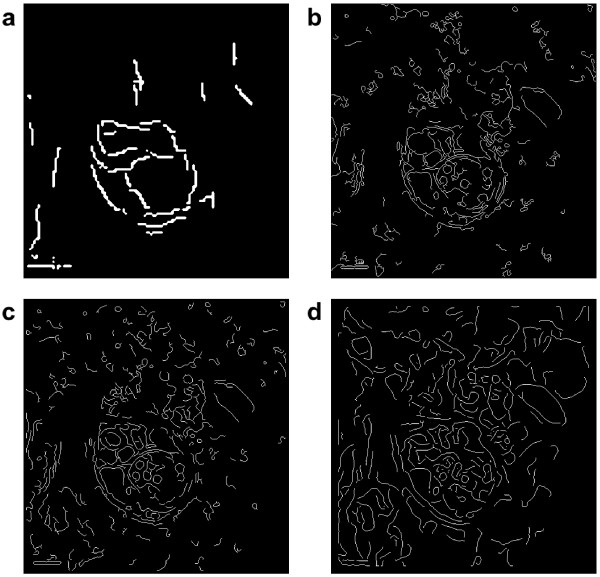
**Edge detection scheme comparison on the vesicle image**. Comparison of different edge detection schemes applied to the vesicle image. a) raw output from out curvelet-based scheme; b) Canny edge detector with thresholds 0.12 and 0.35; c) odd Gabor filter with *σ *= 2 and *ω *= 0.5.; d) odd Gabor filter with *σ *= 6 and *ω *= 0.33.

We apply our method to an electron microscopy image showing a vesicle with some internal structure (see figure 2, top left). Our intention is to find the outer membranes of the vesicle. This task is difficult for several reasons. The image is noisy and full of small structures which makes a direct application of for example a gradient based edge detector useless, since it will detect edges everywhere. Furthermore, smoothing the image will not help much, since it will smooth the thin edges as much as the other structures, making it harder to detect the edges, see figure 3.

Curvelets, provide a multi-scale decomposition of the image that makes it possible to pick just a few scales and ignore for example the finest scale, where most of the noise is, and the coarsest scale, where the large differences in intensity are. Curvelets also provide us with information about directionality in the image, which enables us to search for structures with a strong direction, and trace along them.

For the image in figure 2, we select only level 4 out of the 5 levels of the curvelet decomposition. We then compute the directional field as in section, averaging over the nearest curvelet coefficients in space (index *k*), as well as the nearest curvelet coefficients in direction (index *l*). The magnitude of the field is shown on the top right of figure 2, and a small extract of the image is shown in the middle with the magnitude and direction of the field.

The final result, after applying the non-maximal suppression step on the field data, using weak threshold 0.25 and strong threshold 0.33, and after extending the edges along the directional field to connect the adjacent edge segments, is shown on the bottom right of figure 2, with the edges overlaid in white on the original image. The outer membranes of the vesicle are succesfully detected almost everywhere, and the most prominent internal membranes are also detected. The total execution time was about 1 s on a desktop computer for this image of size 497 × 480, with about half the time being spent on computing the curvelet transform.

**Figure 4:**
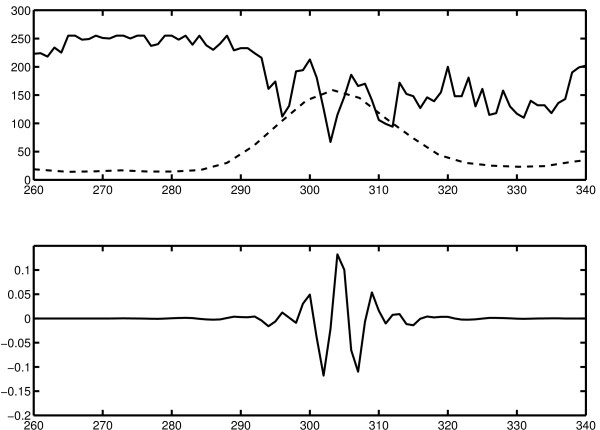
**Cross section of an edge in the image**. Investigation of the cross section of an edge from the image in figure 2. Top: image data from the horizontal line indicated by arrows in figure 2, in the region of the rightmost membrane (solid line), and the curvelet magnitudes on level 4 as extracted by the algorithm (dotted line, arbitrary scale). Bottom: cross section of a curvelet on level 4, approximately aligned with the edge.

In order to better understand which structures are detected by our edge detection scheme, we extract the pixel values along a horizontal line of the image in figure 2, as indicated by the small arrows in that figure. The pixel values in a region near the rightmost membrane of the vesicle are shown as the solid line in the topmost graph in figure 4. The dotted line is the magnitude of the curvelet coefficients at level 4 along the same line as extracted by our scheme. In the bottom graph, the profile of a curvelet on level 4 almost aligned with the edge is shown for comparison. It is clear that the magnitude of the curvelet coefficients is large where the signal matches the shape of the curvelet, which in this case is exactly the area of the double membranes.

We compare our edge detection scheme to the Canny edge detector [4] and the Gabor filter-based edge detector by [5]. In figure 3, we plot the results using the different methods, with two different sizes used for the Gabor filter. The thresholds have been chosen to show the edges of the vesicle clearly, while eliminating the surrounding structures as much as possible. It is clear that our scheme detects the vesicle membranes better than the other two methods. The Canny edge detector and the Gabor filter detector with the smaller *σ *both give multiple responses to the membranes, and detect most of the smaller structures, which makes it hard to distinguish the interesting structures from the background. Using a larger *σ *for the Gabor edge detector eliminates some of the finest structures, but also makes the vesicle membranes harder to detect. No set of Gabor filter parameters was found that produced better results.

### A tube formation assay image

**Figure 5:**
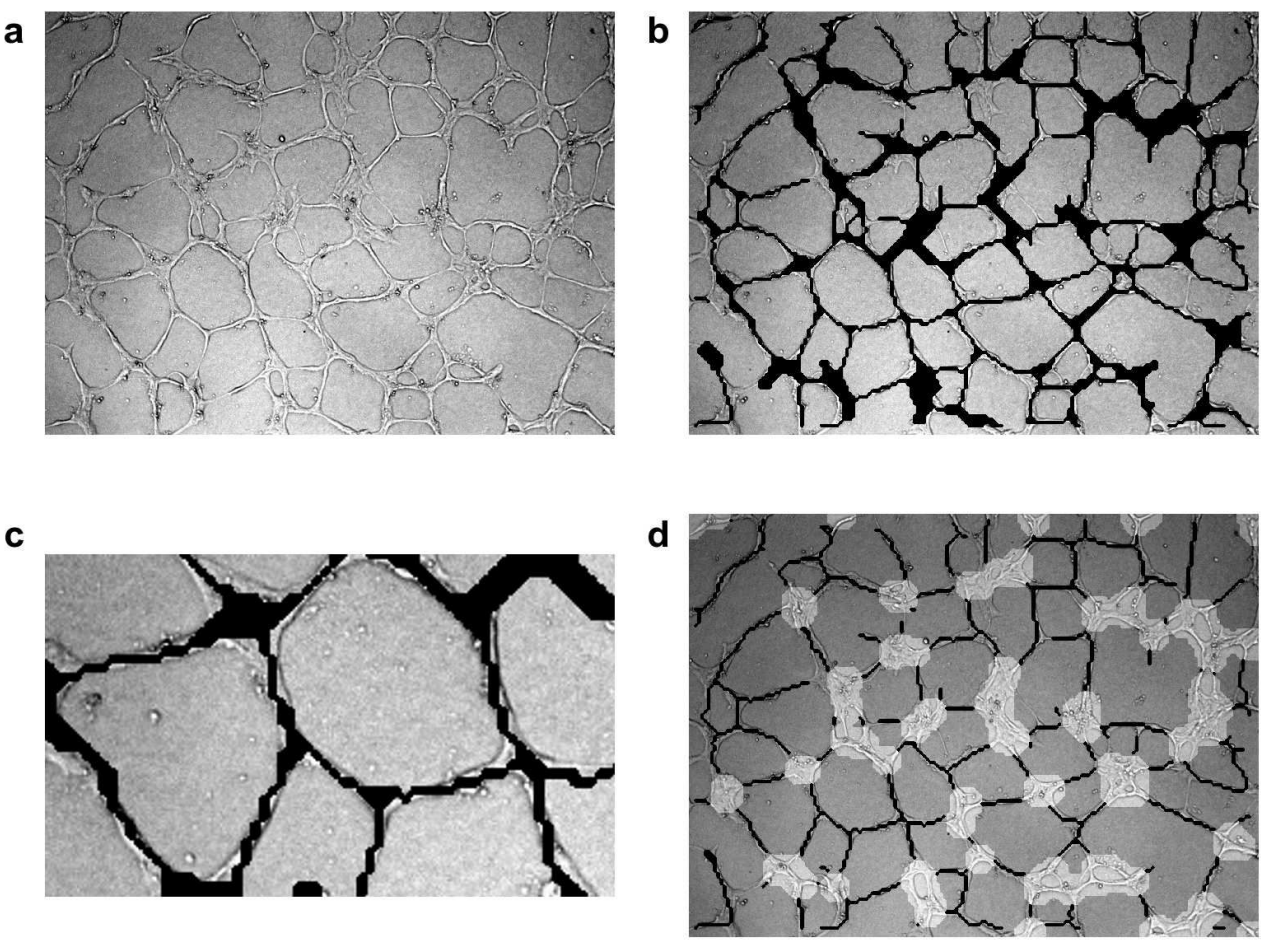
**Edge detection on the tube formation assay**. The edge detection scheme applied to the image on the top left (a) from a tube formation assay, where endothelial cells form vessels (or tubes). The results of the edge detection using levels 4 and 5 of the 6 curvelet levels are shown in (b), with a close-up of a region shown in image (c). For the last image (d), we have also indicated the sheet-like structures, detected as areas where the sum of the curvelet coefficients over all directions is larger than a threshold. Before thresholding, we computed the opening of the image generated from these sums of coefficients to make the detected areas more connected.

As a second example, we apply our method to a light microscopy image from a tube formation assay (see figure 5, top left). In a tube formation assay, endothelial cells are grown on a dish, and their ability to form vessels (or tubes) is investigated by counting the number of tubes seen in the image and computing their length, as well as extracting network information such as the number of junctions. We use this example to see how elongated multicellular structures can be detected using our scheme.

On the top right of figure 5, we see the results from the edge detection scheme overlaid in black on the original image. We have used levels 4 and 5 of the 6 curvelet levels, averaging over two neighboring curvelets in direction and one neighbor in position, and the low and high thresholds 0.22 and 0.31. The edges have then been extended at most 5 steps to connect adjacent edges. Finally, the edges were dilated and then thinned to fill possible holes in the selected areas. An extract from the bottom left of the image is shown on the bottom left of figure 5 to show some more detail. Almost all of the tubes are detected, only a few weaker tubes are not marked. The broader, sheet-like structures are marked because they show internal intensity variations, and in some cases two parallel tubes have been joined together because they can not be distinguished at the detail level we use.

Depending on the desired results, it might be useful to exclude the sheet-like structures from the detection. Increasing the thresholds reduces the detected sheet area, but might also remove some of the tubes. Therefore we use a different method to exclude sheet-like structures. Since the intensity variations in the sheets contain essentially all directions, and not only a few as in the tubes, we use equation (3) to get the total curvelet magnitude at each position and then threshold this information to find the sheets. To further enhance the detection of the sheets, we compute the morphological opening of the total magnitude image using a disk of radius 5 as structural element, before applying the threshold 0.7 times the maximal magnitude of the opened image. The results are shown on the bottom right of figure 5. Here, additionally, the detected tubes have been thinned to show the network structure more clearly. Again some tubes that run in parallel are marked as sheets when they are near each other, and some sheets are not marked as such, but on the whole the distinction between sheets and tubes is clearer than before.

**Figure 6:**
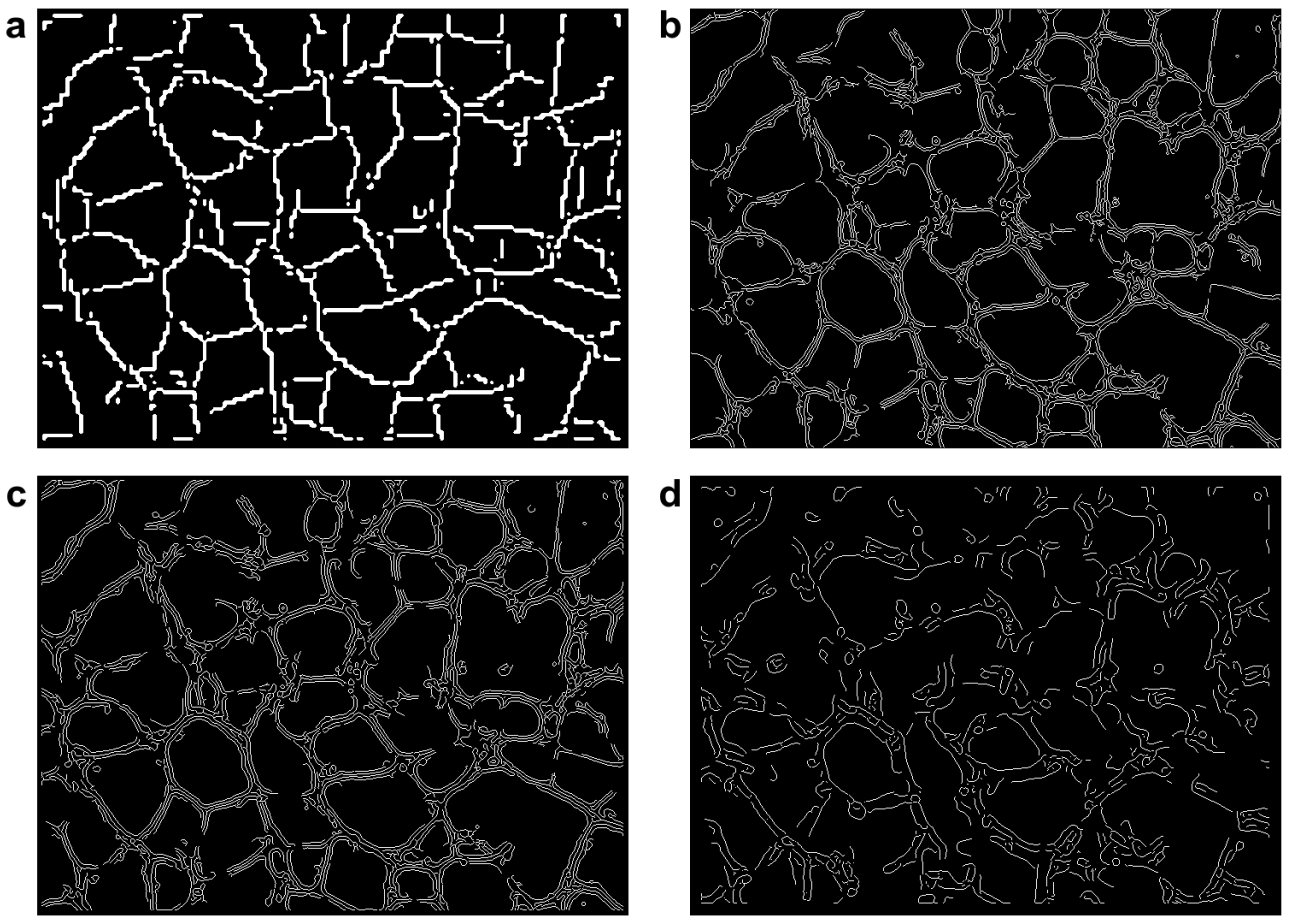
**Edge detection scheme comparison on the tube formation assay**. A comparison of different edge detection schemes for the tube formation image. a) raw output from the thresholding step of our curvelet-based scheme; b) Canny edge detector with thresholds 0.1 and 0.4; c) Gabor filter-based scheme with *σ *= 2 and *ω *= 0.5; d) Gabor filter-based scheme with *σ *= 6 and *ω *= 0.33.

In figure 6, we compare our results for the tube formation image to the results achieved using the Canny edge detector and the Gabor-filter based scheme [5]. We show here the raw output from the thresholding step of our scheme, that is the edges have not been extended as in figure 5. The Canny detector and the Gabor scheme with small *σ *give similar results and detect all the fine edges in the image, thus giving multiple responses to each tube, and also being more sensitive to small structures not belonging to the tube network. It would be possible to process the edge map to get a more connected map of the tubes, also giving a measure of the width of the tubes, which is not easy to get using only our curvelet-based scheme. Our curvelet-based scheme, however, gives a clearer view of the structure of the tube network, and is less sensitive to the small circular artefacts. Using a larger *σ *for the Gabor scheme to smooth the image more gives more of the outline of the tubes, but edges in the resulting edge map often have little in common with actual edges in the image and it is hard to extract the tubes from the edge map.

**Figure 7:**
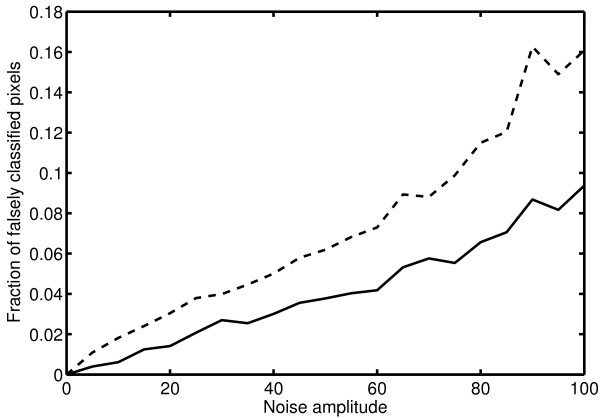
**Sensitivity to noise**. The fraction of falsely classified pixels for images with different levels of added uniform random noise, compared to the edge map computed for the original image in figure 5a. Results are shown for our curvelet-based scheme (solid line) and for the Canny edge detector (dashed line). The analysis parameters were kept the same for all images.

In order to assess the sensitivity of our algorithm to noisy data, we investigate how much the extracted edges change when uniform noise is added to the image. To this end, we added uniform random noise in the interval [-*A, A*] to the image in figure 5a, where *A *varied from 0 to 100 (with original pixel intensity values ranging from 0 to 255). The noisy images were then analyzed by our curvelet-based edge detector and by the Canny edge detector, using the same parameter values as in figure 6 for all images. In figure 7, we show the fraction of pixels in the image that were classified differently in the noisy images compared to the original image. This gives a measure of the sensitivity to noise of the two edge detectors. It is clear that our edge detection scheme is less sensitive than the Canny edge detector, which is to be expected since the noise appears mostly on the finest scale of curvelets, and this scale is ignored in the edge extraction part of our scheme.

## Discussion and conclusion

We have described a method for edge detection based on the discrete curvelet transform, and have seen that it can be useful for finding edges and elongated structures in images where the edges may not easily be detected using traditional methods. The main advantages of the curvelet transform are that it is a multi-scale transform, which enables us to extract edge information from detail levels of our choice and disregard the other levels, and that it gives us directional information at each point which can be used to improve the edge detection. Discarding the information on the coarser levels implicitly performs a background substraction and makes the method insensitive to background intensity variations in the image, while discarding the finest level efficiently excludes most of the noise. Excluding however the finest level also excludes information about edges with the width of a single or very few pixels. Our method should therefore primarily be applied to images where the edges to be detected show variations on a wider scale than only a few pixels.

This is also seen in the precision with which we detect the edges. Since the curvelets are defined on a coarser grid than the original image, the edges we detect will not have pixel precision, but rather the precision of the grid defined by finest selected curvelet level. This makes sense when the edges we search for are several pixels wide.

Since a single edge often leaves a trace on several curvelet levels, and since we may be interested in edges of varying width, it makes sense to include several levels of curvelets in the analysis, and indeed the results often improve when including several levels, as we did in the tube formation example.

When extracting the directional field, we pick the direction with the maximal curvelet coefficient as in (2), using its magnitude as the magnitude of the directional field, since this gives us a measure of the strength of the edge in that particular direction without influence from edges in other directions. It is often a good idea to perform a running average over three or more neighboring directions before picking the maximum, since an edge may have a direction which is not perfectly aligned with a single predefined curvelet direction, in which case it influences the curvelet coefficients for several directions and these should be added to include the entire effect of the edge. By computing the sum over all directions as in (3), we can also detect areas where there are many weaker edges meeting, and by distinguishing these areas from those found by picking the maximum, we can distinguish between isotropic and highly anisotropic areas of the image, as in figure 5, bottom right.

The plots in figure 4 suggest that the curvelet-based edge detection works well when the edges are similar in shape to the curvelet cross section. This is for example the case when the edges we are looking for are double membranes which show up in the image as two parallel dark lines, but also in the second example of the light microscopy image in figure 5, where the tubes have a profile with a bright section in the middle surrounded by darker areas. Our curvelet-based edge detection scheme is not limited to these examples, but is also capable of detecting step edges between bright and dark areas. For these applications, however, the standard edge detection algorithms are faster and more accurate since they work on the single pixel level and are designed for this purpose.

The comparisons to the Canny edge detector and the Gabor filter-based detector in figures 3 and 6 show that our curvelet-based scheme is better at detecting the main structures in the images. Especially for the electron microscopy image in figure 3, our scheme outperforms the other schemes and is capable of separating the vesicle membranes from the background. For the tube formation image in figure 6 the other schemes perform quite well, and the main difference is that our curvelet-based scheme gives the backbone of the network, while not immediately giving the width of the structures. If this is desired, a combination of different edge detection schemes might be an alternative. As shown in figure 7 however, our scheme is less sensitive to pixel noise than the Canny edge detector, which gives an advantage when applying the method to experimental microscopy data.

## Authors' contributions

TG developed and programmed the edge detection method and drafted the manuscript. PK conceived the study, contributed to the development of the edge detection method, edited and finalized the manuscript. All authors read and approved the final manuscript.

## Supplementary Material

Additional file 1 **CurveletUtils.** Matlab source code for a GUI implementing the edge detection method. Click here for file

## References

[B1] Meijering E, van Cappellen G, Shorte SL, Frischknecht F (2007). Quantitative biological image analysis. Imaging cellular and molecular biological functions.

[B2] Carpenter AE, Jones TR, Lamprecht MR, Clarke C, Kang IH, Friman O, Guertin DA, Chang JH, Lindquist RA, Moffat J, Golland P, Sabatini DM (2006). CellProfiler: image analysis software for identifying and quantifying cell phenotypes. Genome Biol.

[B3] Gonzalez RC, Woods RE, Eddins SL (2004). Digital image processing using MATLAB.

[B4] Canny J (1986). A computational approach to edge-detection. IEEE T Pattern Anal.

[B5] Mehrotra R, Namuduri KR, Ranganathan N (1992). Gabor filter-based edge detection. Pattern Recogn.

[B6] Talukder A, Casasent D, Szu HH (1998). Multiscale Gabor wavelet fusion for edge detection in microscopy images. P Soc Photo-Opt Inst.

[B7] Chan TF, Vese LA (2001). Active contours without edges. IEEE T Image Proc.

[B8] Kass M, Witkin A, Terzopolous D (1987). Snakes – active contour models. Int J Comput Vision.

[B9] Candès EJ, Donoho DL (2004). New tight frames of curvelets and optimal representations of objects with piecewise C-2 singularities. Commun Pur Appl Math.

[B10] Candès E, Demanet L, Donoho D, Ying LX (2006). Fast discrete curvelet transforms. Multiscale Model Sim.

[B11] Sivakumar R (2007). Denoising of computer tomography images using curvelet transform. ARPN J Eng Appl Sci.

[B12] Dettori L, Semler L (2007). A comparison of wavelet, ridgelet, and curvelet-based texture classification algorithms in computed tomography. Comput Biol Med.

[B13] Bermejo-Moreno I, Pullin DI (2008). On the non-local geometry of turbulence. J of Fluid Mech.

[B14] Daubechies I (1992). Ten lectures on wavelets, of CBMS-NSF conference series in applied mathematics.

